# Research designs to generate evidence of HIV post‐exposure prophylaxis effectiveness for new long‐acting agents

**DOI:** 10.1002/jia2.26475

**Published:** 2025-06-26

**Authors:** Katrina F. Ortblad, Elizabeth R. Brown, Renee Heffron, Kenneth Ngure, Andrew Mujugira, Deborah Donnell

**Affiliations:** ^1^ Public Health Sciences Division Fred Hutchinson Cancer Center Seattle Washington USA; ^2^ Vaccine and Infectious Disease Division Fred Hutchinson Cancer Center Seattle Washington USA; ^3^ Department of Biostatistics University of Washington Seattle Washington USA; ^4^ Heersink School of Medicine University of Alabama at Birmingham Birmingham Alabama USA; ^5^ School of Public Health Jomo Kenyatta University of Agriculture and Technology Nairobi Kenya; ^6^ Department of Global Health University of Washington Seattle Washington USA; ^7^ Infectious Disease Institute Makerere University School of Public Health Kampala Uganda

**Keywords:** effectiveness, HIV, HIV prevention, long‐acting prophylaxis, post‐exposure prophylaxis, study designs

## Abstract

**Introduction:**

New longer‐acting antiretroviral (ARV) drugs—that is single doses with antiviral activity for at least a month—are being utilized for HIV treatment and pre‐exposure prophylaxis (PrEP) but have not been explored for post‐exposure prophylaxis (PEP). A “one‐and‐done” simplification of PEP has the potential to serve the HIV prevention needs of individuals not being met with traditional services and expand overall biomedical HIV prevention coverage. We discuss challenges with the assessment of PEP effectiveness in human trials and potential study designs that could generate evidence needed to inform the use of new, single‐administered, long‐acting ARVs for PEP.

**Discussion:**

Challenges with determining the effectiveness of new long‐acting PEP agents in human trials include the low likelihood of observing an HIV acquisition and the short period for outcome assessment (likely 1 month) following PEP administration. Additional challenges include recruiting individuals in the brief window in which they could benefit (<72 hours of a potential HIV exposure) and ethics of conducting informed consent during a period of high stress/vulnerability. Consequently, design approaches where the efficacy goal is to establish that the HIV incidence rate following PEP administration (of the standard or a novel agent) approaches zero should be considered. HIV RNA testing conducted within 5 days of a potential exposure could define prevention per exposure. Novel recruitment venues—such as community‐based retail or online pharmacies—could be used to reach individuals after a potential exposure. Potential study designs include one‐ or two‐arm individual‐level product assignment aimed at demonstration of short‐course efficacy or longer‐term effectiveness compared to a background rate; cluster‐randomized controlled trials of recruitment venues; and novel individual‐level approaches that either do not or do utilize randomization in combination with choice, enabling assessment of preferences and effectiveness.

**Conclusions:**

Over the past decade, multiple new HIV PrEP products—but no new PEP products—have been developed to meet the diverse needs of individuals seeking HIV prevention services. Challenges exist with generating PEP effectiveness evidence, but they are not insurmountable. Effectiveness research on new PEP products could advance the number of HIV prevention options available.

## 1 INTRODUCTION

New long‐acting antiretroviral (ARV) drugs—in the form of 2‐monthly injections, semiannual injections and monthly pills—are now available or in late‐stage development as HIV treatment and/or pre‐exposure prophylaxis (PrEP) use [1−3]. These drugs additionally offer a potential “one‐and‐done” approach to HIV post‐exposure prophylaxis (PEP), a potential simplification of the current daily oral regimen, but it remains unclear what would be required to establish them as effective for PEP use.

Most current PEP guidelines recommend a daily, oral three‐drug ARV regimen for 28 days [4]. This recommendation is based on extrapolations from HIV treatment studies demonstrating superior halting of HIV disease progression and reduced risk of drug resistance with three‐ versus two‐drug regimens [5], as well as the availability of daily co‐formulated three‐drug pills [6]. However, poor drug tolerability and low completion rates have challenged the implementation of three‐drug PEP regimens [7]; especially regimens containing protease inhibitors, which result in side effects (∼70%) and premature course completion (∼30−50%) for many users [8, 9]. Further, despite evidence in animal data on the increased safety and potency of three‐ versus two‐drug PEP regimens [10], associated laboratory testing requirements and dosage recommendations based on two‐drug regimens have not been simplified for three‐drug regimens.

While PEP has been available as an HIV prevention tool for more than three decades, it remains underutilized and understudied. Since ARVs were demonstrated as effective for PrEP and approved in 2012 [11], most HIV programmes have focused on oral PrEP delivery and reserved PEP for emergency situations (e.g. occupational HIV exposures, instances of sexual assault). Global PrEP dispensing is carefully monitored and complimented with metrics of success; no such monitoring or metrics exist for PEP [6]. Meanwhile, the promise of PrEP as an HIV prevention tool has been challenged by poor uptake and adherence; individuals struggle with daily pill taking, cycle in and out of periods of PrEP need, and cannot always anticipate encounters associated with HIV risk [12, 13]. Consequently, some individuals may prefer PEP over PrEP for their HIV prevention needs. Recent implementation studies have found that when PEP is co‐delivered as an HIV prevention option with PrEP, PEP uptake is high and PEP‐to‐PrEP transition low [14−17]. Many preference studies have demonstrated the appeal of long‐acting ARVs to PrEP users [18−22]; these long‐acting agents are likely to also appeal to PEP users.

In this paper, our intent is to present conceptual approaches for a PEP efficacy or effectiveness study, rather than concrete trial designs; recognizing the importance of collaboration with regulatory authorities, clinicians, biostatisticians, ethicists and community members to select and develop a specific design. Additionally, we outline design challenges with PEP effectiveness research and consider possible settings for implementation. In these studies, we assume the goal is to establish evidence that a selected long‐acting ARV—that is a single dose with a dosing interval of at least a month—has comparable effectiveness to the current three‐drug PEP regimen, the standard‐of‐care (SOC). A long‐acting PEP regimen has strong potential to overcome uptake and adherence obstacles faced by the daily SOC—as demonstrated with PrEP [1−3], simplify PEP implementation and increase the number of HIV prevention options avaliable.

## 2 DISCUSSION

### 2.1 Challenges with assessing PEP effectiveness

To assess effectiveness of new long‐acting ARVs for PEP use in a rigorous human trial, several design challenges exist (Table 1). First, the likelihood of an observed HIV infection following PEP initiation is low because many HIV exposures that trigger initiation are not true exposures (i.e. the exposing partner is not living with HIV); many true exposures may be associated with low or no HIV transmission risk (if the exposing partner's HIV viral levels are low or undetectable). While the potential effectiveness of a new long‐acting PEP agent is likely high—as is the existing PEP SOC, it is unknown whether long‐acting systemic ARV formulations are absorbed quickly enough to prevent HIV acquisition post‐exposure.

**Table 1:** Design challenges with demonstration of PEP effectiveness in a human trial

Challenge	Details
**Exposure to HIV unknown**	Self‐reported potential HIV exposures result in uncertainty around whether the encounter was a “true” exposure (i.e. the exposing partner may not be living with HIV)
**Undetectable = Untransmissible**	Even after a “true” HIV exposure to an individual living with HIV, there may be no or low‐risk transmission if the exposing partner is HIV virally suppressed or on the way there.
**High potential effectiveness of new long‐acting agent**	If a new long‐acting PEP agent is highly effective, then the risk of HIV acquisition and chance of observing the primary endpoint (i.e. incident HIV infection) is very small.
**Speed of drug absorption for new agents unknown**	Uncertainties exist around how rapidly new long‐acting ARVs are absorbed, which may present challenges for using these agents as PEP; however, these remain hypotheses.
**Unknown SOC PEP effectiveness**	Current PEP guidelines are based on animal model data and observational cohorts; no robust effectiveness evidence from human trials exists.
**Evaluation period for effectiveness short**	Since the potential HIV exposure occurred in the past, the follow‐up duration for an observed event (i.e. HIV infection) is short, likely only 1 month or potentially shorter to control for potential new HIV exposures.
**PEP demand unknown**	Historic restrictions on PEP access and limited data on PEP dispensing result in uncertainties around PEP demand; which could challenge the recruitment of potential trial participants, especially during the brief window of PEP effectiveness.
**Operational speed of PEP delivery critical**	PEP effectiveness is greatest within 24 hours and up to 72 hours of HIV exposure; novel PEP delivery models are needed to reach individuals within that window.
**Conducting informed consent**	The time‐sensitive nature of the intervention, lack of effectiveness evidence for the SOC and ethical considerations present challenges for informed consent.
	

Abbreviations: ARV, antiretroviral; PEP, post‐exposure prophylaxis; SOC, standard‐of‐care.

Other design challenges include the unknown effectiveness of the current PEP SOC and the short evaluation period for PEP effectiveness following a potential HIV exposure. The current PEP SOC effectiveness data comes from animal models and observational cohorts [10, 23−26]; no powered human efficacy trials have ever been conducted. Additionally, the evaluation period following a potential PEP exposure is short—likely only 1 month—compared to a PrEP or treatment effectiveness trial, where participants can be evaluated for months or years. The SOC PEP agent recommends consistent daily dosing for 28 days—to maintain systemic levels of active drugs during the initial HIV replication and infectivity period—with HIV testing at the end of this period [4]. With novel agents that are administered once and maintain high systemic levels for a month or longer, it may be important to test for HIV sooner. The opportunity for additional HIV exposures following PEP administration also increases with time (e.g. through sexual activity, injection equipment sharing), making it difficult to determine whether an HIV acquisition is a result of PEP failure or new exposure.

Recruitment for a PEP effectiveness trial may also be challenging as the demand for PEP remains largely unknown, the window in which individuals could benefit from PEP is brief and ethical considerations may restrict the populations considered for recruitment. In many settings, PEP is not widely available [27] and access is restricted to individuals who have experienced an occupational HIV exposure or sexual assault. Thus, the feasibility of accessing potential PEP clients and engaging them in clinical research during the acute period in which PEP could demonstrate effectiveness—that is within 72 hours, and ideally 24 hours, of a potential exposure [28]—remains unknown. To help ensure PEP reaches individuals in the window of benefit, innovative delivery models that facilitate quick PEP access are necessary [29]. Quick PEP delivery, however, may complicate the informed consent process—which is necessary for joint decision‐making around the use of a novel agent compared to the SOC, especially an SOC that lacks rigorous effectiveness evidence [6]. To facilitate consent during a moment of potential high stress and uncertainty, researchers could consider strategies utilized to consent survivors of psychological trauma or sexual assault—such as having trained counsellors on site to support consenting participants or assuring participants of their autonomy throughout the process [29, 30].

Finally, another challenge with assessing PEP effectiveness—outside of those related to study design—is obtaining funding for a human trial; which is likely to be expensive and unlikely to be commercially viable for pharmaceutical companies.

### 2.2 Settings and populations for PEP effectiveness trials

To reach individuals who could benefit from PEP within 72 hours of an exposure associated with HIV risk, strategic recruitment venues (with capacity for ARV dispensing) in HIV endemic settings need to be identified to achieve sample sizes sufficient to demonstrate PEP effectiveness. Established healthcare facilities—which were successfully utilized as recruitment venues for PrEP efficacy trials [31]—may be poor recruitment venues for PEP efficacy trials since they often are far from where individuals live, overcrowded and have limited hours of operation [12, 32−35]. Rather, recruitment venues that are community‐based (i.e. outside healthcare facilities and near to where people live), located near hotspots of sexual activity and open on weekends/evenings when HIV exposures are more likely to occur may be potentially more high‐yield for PEP trials.

Examples of such community‐based venues could include retail pharmacies—especially those located near universities or bars—and online pharmacies that offer on‐demand telehealth visits followed by quick courier‐delivered services. Recent implementation studies that delivered PEP and PrEP via these novel delivery platforms in Kenya demonstrated high PEP uptake; with 68% (3228/4772) of retail pharmacy PrEP/PEP clients [36] and 88% (1549/1754) of online pharmacy PrEP/PEP clients [17] initiating PEP. These venues are settings where individuals seek other sexual and reproductive health services, including emergency contraception—which is sought more frequently at retail pharmacies than at public clinics in Kenya [37]; engaging such clients could facilitate PEP reach to individuals who might not otherwise engage. However, implementation challenges for delivering long‐acting PEP products in community settings would need to be addressed, such as cold chains, reliable electricity and the delivery of HIV RNA testing [38].

In many PEP implementation studies and programmes, transitioning clients from PEP to PrEP has remained a persistent challenge. In studies that implemented strategies to support PEP‐to‐PrEP transition, <50% of PEP clients (between 11% and 49%) later initiated PrEP [39]. This evidence suggests that some individuals might only have periodic HIV exposures better suited for PEP use or that some individuals might prefer repeat PEP use over continuous PrEP use as a long‐term HIV prevention strategy—a preference we could leverage when designing PEP effectiveness trials [40−42].

### 2.3 Potential trial approaches for assessment of PEP effectiveness

The ultimate trial endpoint to determine the efficacy of a novel long‐acting PEP agent is incident HIV infection. Assessing this endpoint soon after PEP administration may be necessary to avoid the potential for new HIV exposures. HIV RNA testing can detect the virus within 5 days (interquartile range 3.1−8.1 days) of a potential exposure [43]. Thus, a study endpoint using HIV RNA testing could commence as soon as 1‐week post‐treatment, with additional assessments up to 1 month to verify no infection occurred. However, few HIV acquisitions are likely to be observed in a PEP effectiveness trial; which makes a traditional, fully powered non‐inferiority trial comparing incidence rates between two groups infeasible. Smaller trials would be possible if we reached a consensus about: (1) whether the “efficacy” goal is to establish that the probability of HIV infection following PEP administration (of the SOC or a long‐acting agent) is sufficiently low; and (2) what threshold would be considered sufficiently low.

Design approaches where the “efficacy” goal is to establish that the rate of HIV acquisition following PEP administration approaches zero should be considered. Borrowing from contraceptive efficacy trials [44], this could be based on a consensus‐based, evidence‐driven threshold and the trial powered on the upper bound 95% confidence interval for the probability of HIV acquisition following PEP administration. To address challenges with low observed HIV acquisition rates in PrEP efficacy trials, statistical methods were developed to estimate background HIV infection rates [45, 46]; PEP efficacy trials would benefit from the development of similar methods with the difference being that these would likely need to focus on exposure‐level versus cumulative HIV incidence rates.

Retaining incident HIV infection as the endpoint, we have identified five potential study design concepts that could be utilized to demonstrate the efficacy or effectiveness of a long‐acting PEP agent in a human trial (Table 2). All the designs proposed could aim to compare HIV incidence rates between two groups or demonstrate an HIV incidence rate approaching zero—or a consensus‐based threshold—in one or more groups with proven HIV acquisition risk.

**Table 2:** Potential designs for demonstration of PEP effectiveness in a human trial

Description	Population	Outcome	Estimand	Advantages	Challenges	Example designs
**1. Individual‐level randomization: short‐course efficacy *(per exposure)***						
Randomize individuals; follow‐up for 1–6 months	Individuals seeking PEP	HIV infection^a^ at 1 month (primary) and when LA agent no longer active (secondary)	Proportion with HIV in each study group	Can demonstrate per exposure efficacy	Depends on fixed or background HIV incidence rate Difficult to power for comparison of different PEP agents Need to consider returning participants (re‐randomize?^b^)	Emergency contraception [47] COVID [48]
**2. Individual‐level randomization: long‐term effectiveness *(repeated exposures using PEP)***						
Randomize individuals; follow‐up for a pre‐specified trial duration	Individuals seeking PEP (or with PEP use history) interested in a PEP‐based HIV prevention strategy	HIV infection^a^ (frequency of testing TBD)	Proportion with HIV in each study group; after a pre‐determined follow‐up time	Likely pragmatic design Adequate statistical power more likely since assessing effectiveness (i.e. dependent on user compliance) versus efficacy	Recruitment of clients interested in repeat PEP use as an HIV prevention strategy Participants may drop out differentially if they dislike their assigned PEP regimen	On demand PrEP [49] Doxy PEP [50, 51]
**3. Cluster‐level randomization**						
Randomize delivery settings (i.e. pharmacies, clinics)	Clients of delivery settings/recruitment venues with high PEP dispensing	HIV infection^a^ (individual level)	Proportion with HIV in each study group; after pre‐determined follow‐up time (cluster adjusted)	Simpler implementation at site level	Participants may select sites that offer their preferred agent, resulting in potential contamination High implementation costs Complicated logistics Large sample size needed	Pharmacy PrEP/PEP delivery [52] Leprosy [53]
**4. Individual‐level, no randomization: 100% choice**						
Allow individuals to choose the SOC or LA PEP agent	People seeking PEP	HIV infection^a^	Proportion with HIV in each study group	No ethical concerns Participants receive preferred PEP agent	If choice associated with the probability of true HIV exposure, observed differences will be confounded with choice	Open‐label extension studies for CAB‐LA versus FTC/TDF [54] Cross‐over study designs with choice period [55]
**5. Individual‐level, hybrid randomization and choice: preference design**						
Allow individuals to choose their PEP product (SOC or LA agent) or choose to be randomized (SOC vs. LA agent)	People seeking HIV PEP	HIV infection^a^	Proportion with HIV in each study group	Can elucidate efficacy of different interventions and impact of product preference and assignment on effectiveness	Accounting for differences in product choices based on product characteristics Difficult to plan power when proportion willing to be randomized unknown.	Never been done in HIV literature; other examples include: Trials for mental health or addiction treatment [56]

Abbreviations: CAB‐LA, long‐acting injectable cabotegravir; FTC, emtricitabine; LA, long‐acting; PEP, post‐exposure prophylaxis; PrEP, pre‐exposure prophylaxis; SOC, standard‐of‐care; TDF, tenofovir disoproxil fumarate.

^a^ All incident HIV infections would ideally be confirmed with HIV RNA testing.

^b^ Re‐randomization of returning participants could be challenging if they return within the window of effectiveness for the long‐acting PEP agent.

The first two individual‐level trial designs propose randomizing individuals seeking PEP to either the SOC or a new long‐acting agent. The first individual‐level trial design is focused on the demonstration of short‐term per‐exposure PEP efficacy. In this design, individuals are followed for one PEP course and the proportion that acquired HIV in each group is assessed at a fixed duration following PEP administration. There are several options for follow‐up in this design. In the simplest approach, participants are followed for 1 month and eligible for PEP administration once. In a more complicated approach, participants can repeat PEP use (outside the window of the long‐acting agent's potential effectiveness) and be re‐randomized or re‐dosed. Because the risk of HIV acquisition with either PEP agent is likely near zero, an evaluation against a consensus‐based threshold, rather than a two‐group comparison, would be suggested.

The second individual‐level trial design focuses on the demonstration of the effectiveness of repeat PEP use over time. For this design, individuals interested in a PEP‐based HIV prevention strategy would be recruited and followed for a pre‐determined observation period (longer than 1 or 6 months). With this approach—which would likely require a pragmatic, unblinded design—different effectiveness may occur due to differences in the uptake of and adherence to the two PEP agents over a longer observation period, increasing the feasibility of achieving sufficient statistical power between the two groups due to higher resulting HIV incidence rates. Challenges, however, would include the recruitment of clients interested in repeat PEP over PrEP use as an HIV prevention strategy and potential differences in loss‐to‐follow‐up within groups based on clients’ satisfaction with their assigned PEP agent.

The next potential design is a cluster‐level trial, with randomization of high‐yield PrEP dispensing locations (i.e. retail pharmacies) to deliver either the SOC or long‐acting PEP agent. With this design, we could compare the proportion of participants in each study group who acquired HIV after a pre‐determined study period and adjust for clustering. The advantages of this design would be that implementation is simpler at a site versus individual level. Challenges, however, would be the high implementation costs and complicated logistics associated with recruiting, consenting and monitoring the large sample size required for a cluster‐level trial. A potential risk of this design is contamination between study groups if participants select sites that offer their preferred PEP agent.

The final two individual‐level designs would integrate individuals’ preferences for HIV prevention products into our PEP effectiveness assessment. In the first of these designs, there would be no randomization, and individuals seeking PEP could either select the SOC or long‐acting agent after being given sufficient information on both. The advantages of this approach are that there are no ethical concerns and participants receive their preferred PEP agent; challenges arise, however, if participants’ choice is associated with their per‐exposure HIV acquisition risk, which might confound observed differences in HIV incidence rates between the study groups. In the second of these designs, a preference trial, individuals seeking PEP could choose to either receive their preferred PEP agent or, if they did not have a preference, be randomized to one of two agents. Advantages with this approach are the ability to assess the impact of product preference and assignment on PEP effectiveness, while challenges include accounting for differences in choices made because of product characteristics and the need for a large sample size to accommodate the (unknown) proportion unwilling to be randomized.

In all these designs, understanding and supporting the validity of a choice to repeat PEP rather than convert to ongoing PrEP, would need to be explored. There is little data on the demand/need for repeated PEP use and little understanding of whether oral on‐demand PrEP (with a 2‐1‐1 schedule) [49], or PrEP itself, is utilized in practice as PEP. One possible approach is for each enrolled participant to be offered, following a PEP “event,” the opportunity to transition to PrEP, access on‐demand PrEP (for eligible, male participants) or repeat PEP use. Then, participants who do not choose a PrEP option could contribute evidence of PEP use at future times of potential HIV exposure. For each new exposure, repeated or cross‐over randomization—which allows for allocation to different PEP agents at each event—could be considered. While long‐acting PEP agents would complicate the repeated randomization approach, the prospect of experiencing different products may appeal to participants and enable the collection of preference data based on user experience.

We acknowledge the significant challenges in developing approaches to assess the efficacy or effectiveness of long‐acting PEP agents. After all, the current SOC PEP agent was not based on a human efficacy trial and there are only a few examples of trials evaluating PEP efficacy [57, 58]. Nonetheless, the considerable promise of a long‐acting PEP agent justifies a creative, collaborative effort to develop an approach to establish the effectiveness of this new potential HIV prevention tool.

## 3 CONCLUSIONS

For decades, HIV prevention programmes were limited by the interventions (biomedical and other) and implementation strategies that supported their delivery. With the availability of new long‐acting ARVs, we have entered a new era of choice in HIV programming. As we move forward with multiple PrEP products and options for HIV prevention, new opportunities to enhance and simplify PEP services are needed. One‐time PEP use facilitated with long‐acting ARVs could be a game‐changer for individuals who struggle with daily pill taking, have challenges anticipating encounters associated with HIV exposure and value discretion with the use of a biomedical HIV prevention product. While challenges may exist to the generation of such effectiveness evidence, that should not prevent the exploration of paths forward. To end the AIDS epidemic, we need to engage individuals whose needs are unmet by existing HIV programmes; expanding the number of PEP options available will help achieve this objective.

## COMPETING INTERESTS

The authors have no competing interests to disclose.

## AUTHORS’ CONTRIBUTIONS

The topic of this commentary was developed by DD with authors KFO, RH, AM, KN and ERB contributing to the generation of ideas included in the manuscript. KFO and DD drafted the first version of the manuscript with input from authors RH, AM, KN and ERB. All authors edited the manuscript and approved the final version for publication.

## FUNDING

The authors received no specific funding for the development of this manuscript. KFO and RH were supported by the US National Institute of Mental Health (R00 MH121166, K24 MH123371).
